# The impact of integrated health Qigong and dance exercise on cardiovascular function in middle-aged and elderly women

**DOI:** 10.1097/MD.0000000000040040

**Published:** 2024-10-18

**Authors:** Fan-Wen Zeng, Qian-Yi Zhang, Wei-Jie Zong, Xiang-Ji Peng, Hui Yang

**Affiliations:** a Physical Education College of Jiangxi Normal University, Jiangxi, China; b Chinese Exercise for Life Enhancement Division, Beijing Sport University, Beijing, China; c Wushu Routine Teaching and Research Department, Beijing Sport University, Beijing, China.

**Keywords:** cardiovascular function, health Qigong, middle-aged and older women, randomized controlled trial, traditional exercise

## Abstract

**Background:**

The aim of this study was to evaluate the impact of health Qigong on vascular elasticity, blood lipid levels, and cardiac function in middle-aged and elderly women. By comparing various indicators preintervention and postintervention, the research provides valuable insights into the effectiveness of health Qigong in enhancing cardiovascular health within this demographic.

**Methods:**

A total of 40 middle-aged and elderly women were randomly assigned to 2 groups. The experimental group, consisting of 20 women, practiced health Qigong combined with Tibetan dance for 12 weeks, 3 times per week, with each session lasting 60 minutes. The control group, also consisting of 20 women, continued their regular routines without any exercise intervention. Cardiovascular function metrics were subsequently compared between the 2 groups.

**Results:**

(1) Pulse wave velocity: in the experimental group, significant improvements were observed, particularly in the right ankle (*P* =.02 for left ankle, *P* =.00 for right ankle). The control group showed no significant differences (*P* =.08 for both ankles); (2) blood lipid levels: the experimental group demonstrated significant reductions in total cholesterol and triglyceride levels (*P* =.00 for both), while the control group showed no significant changes (*P* =.59 for total cholesterol, *P* =.71 for triglycerides). There were significant differences in high-density lipoprotein levels between the experimental and control groups (*P* =.00 and .01, respectively); (3) cardiac function: significant improvements were noted in cardiac output (Teich) and stroke volume (Teich) in the experimental group (*P* =.00 for both), while the control group showed no significant differences (*P* =.71 for cardiac output, *P* =.06 for stroke volume).

**Conclusion:**

Health Qigong, integrated with dance exercise effectively enhances pulse wave velocity, blood lipid levels, and cardiac function in middle-aged and elderly women. These findings suggest that incorporating such exercises may contribute to the prevention or delay of atherosclerosis and cardiovascular disease in this population.

## 1. Introduction

Cardiovascular function is a critical indicator of cardiovascular disease and a key marker of aging. Throughout life, the heart and blood vessels work continuously, but as people age, their physical functions inevitably decline, and the incidence of cardiovascular diseases tends to increase among younger populations. These diseases significantly impact the physical and mental health and daily lives of middle-aged and elderly patients, adding pressure to the medical industry.^[1]^ According to the China Cardiovascular Health and Disease Report 2021, the prevalence of cardiovascular diseases in China is rising, with an estimated 330 million people affected.^[2]^ Hypertension is a major contributor to this issue, and the survey indicates that hypertension prevalence is slightly higher in women than in men, highlighting the need for special attention to the cardiovascular health of mature women.^[3]^ Traditional physical exercise has beneficial effects on the physiology, biochemistry, physical function, quality of life, sleep quality, and alleviates depression in patients with cardiovascular disease.^[4–7]^ Regular physical activity, particularly aerobic exercise, can lower the resting heart rate of the elderly, reduce sympathetic excitability, and regulate the heartbeat, thereby improving myocardial contractility and reserve capacity, effectively preventing and controlling heart conditions like tachycardia.^[8]^

Health Qigong is a traditional Chinese practice that focuses on regulating the body, breath, and mind. Evidence-based research classifies health Qigong as a moderate-intensity aerobic exercise, effective in maintaining and promoting cardiovascular health.^[9–12]^ However, most previous studies have focused on single sets of health Qigong exercises, lacking targeted actions for specific diseases. This study incorporates a variety of health Qigong exercises, including *Ba Duan Jin*, *Wu Qin Xi*, *Da Wu*, *Ma Wang Dui*, and *Shi Er Fa*, along with basic movements and it includes Tibetan dance movements. These exercises emphasize spinning and winding pressure actions, meridian dredging, small artery dilation, spasm relief, and improved blood circulation to prevent hypertension and resulting coronary heart disease. Additionally, the aim of this study is to diversify health Qigong, offering new forms of exercise to provide practitioners with more options and enjoyment.

## 2. Materials and methods

### 2.1. Participants

To investigate the effects of health Qigong combined with dance practice on vascular elasticity, blood lipid levels, and cardiac function in middle-aged and elderly women, a random sampling was conducted among members of the Beijing Haidian District Health Qigong Association and nearby communities around Beijing Sports University. The study specifically targeted women aged 45 to 70 years who had no serious cardiovascular diseases and could complete the entire experiment. Forty women were recruited based on the inclusion criteria, their willingness to participate, and consent to sign the informed consent form. All participants were informed about the experimental content, process, and necessary precautions before the experiment. The research was approved by the Experimental Ethics Committee of the Sports Science Department of Beijing Sports University (approval number: 2021051H).

### 2.2. Inclusion and exclusion criteria

The inclusion criteria were: (i) women aged 45 to 70 years; (ii) women capable of actively cooperating with the study and possessing some exercise capacity; (iii) women who had not participated in other exercise programs for approximately 3 months; and (iv) women who could commit to participating in the entire course of the experiments and adhere to the study’s provisions.

The exclusion criteria were: (i) women with conditions such as hypertension, diabetes, hypercholesterolemia, severe cardiovascular, and cerebrovascular diseases; (ii) women intending to join other exercise training programs during the study period; and (iii) women with abnormal thyroid function, bone and joint issues, or other conditions affecting their ability to exercise.

### 2.3. Intervention content

**Figure 1.**
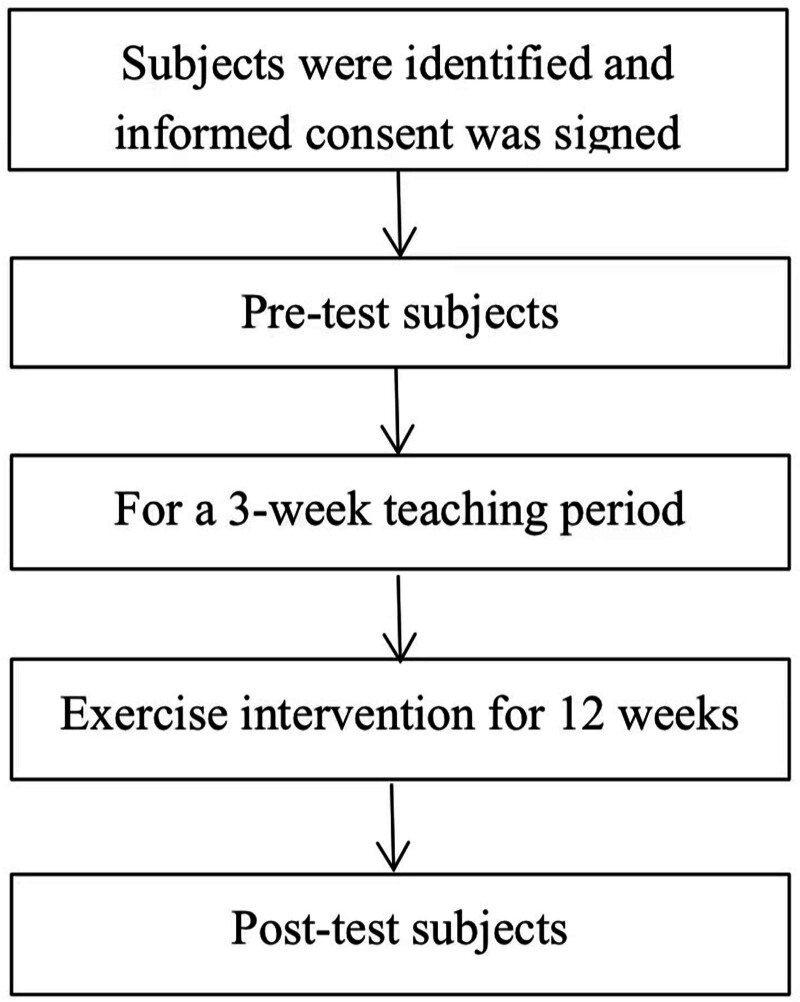
Experimental flow chart.

The experimental group:

Following the pretest, participants underwent a 3-week instructional period. The aim of this period is to master the complete set of movements for health Qigong combined with Tibetan dance. This enabled them to practice independently to music under teacher guidance, ensuring they met the experiment’s basic requirements. The program intervention spanned 12 weeks, with training conducted 3 times weekly at the same location and led by multiple instructors.^[13–16]^ Each session lasted 60 minutes. A posttest was administered immediately after the formal intervention exercises. Figure 1 shows the specifics of the experiment.

Each course was arranged as follows:

- Training location: Daoyin Health Center of Beijing Sports University.
- Training time: 14:00 to 15:00 on Monday, Wednesday, and Saturday.
- Intervention period: 12 weeks.

Training schedule (Table 1):

**Table 1:** Course arrangement.

Intervention process				
Course part	Action name	Groups	Times	Rest
Ready to part	Joint winding, stretching and other actions	Warm up 10 minutes		
Base component	Health Qigong combined with Tibetan dance	5 min/each time, 2 times/group	3 groups	5 minutes
Latter end	Muscle massage, flapping and other movements	Relax 10 minutes

- Preparation: warm up for 10 minutes.
- Basic part: under the supervision of the researcher, participants practiced each movement to music, with each movement session lasting 5 minutes, totaling 40 minutes.
- End: relax and stretch for 10 minutes.

**Figure 2.**
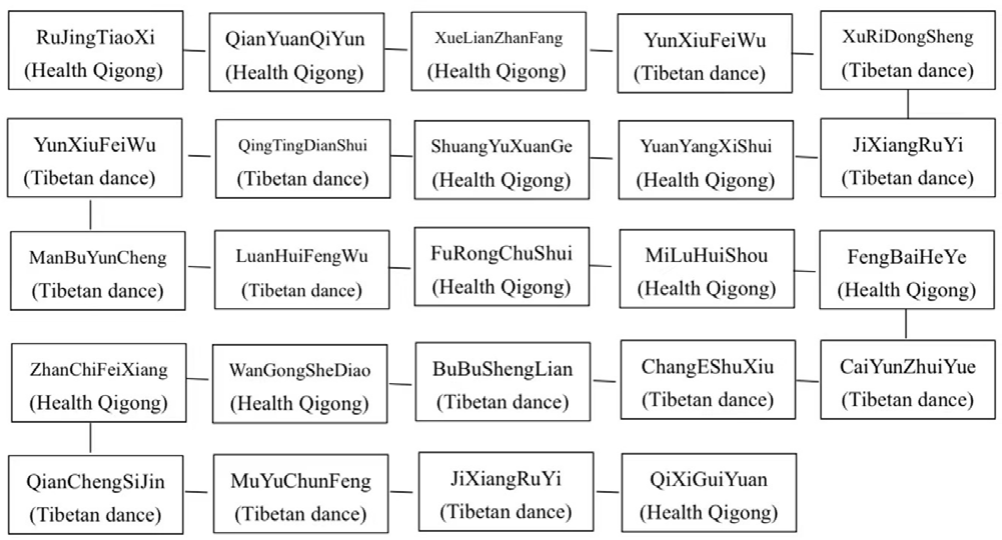
Action name and flow chart.

The complete set included 24 movements (see Fig. 2), accompanied by music. The exercises were conducted at a moderate intensity, aiming to keep the heart rate between 100 and 135 beats per minute.

The control group received no exercise intervention and were instructed to continue their regular routines. These participants were unaware of the proposed intervention and only underwent basic data collection, along with pre- and post-experimental tests. The pretests were conducted on July 2, 2019, and the posttests on October 22, 2019, using identical testing instruments and conducted by the same testers.

### 2.4. Control of irrelevant variables

Participants were divided into the control group and experimental group using simple randomization. They were assigned random numbers, with odd numbers designated to the experimental group and even numbers to the control group. Regarding blinding, the researchers conducting the intervention were not involved in evaluating the indicators. The evaluators were unaware of both the group assignments and the participants’ intervention status.

The tests were conducted in a dedicated testing facility using standardized instruments overseen by a designated tester. Throughout the intervention, trained staff provided exercise guidance and meticulously recorded attendance. Participants refrained from engaging in additional physical exercise, interventions, or psychological counseling that could potentially influence test outcomes and compromise the experiment’s scientific validity. Data was accurately entered into designated fields, with original data being securely backed up.

### 2.5. Test tools

Cardiac output (CO) and stroke volume (SV) (Teich) were assessed using echocardiography conducted by a resident physician in a specialized echocardiography room, utilizing a Philips IE33 color tissue Doppler ultrasound diagnostic instrument. For the pulse wave velocity (PWV) assessment, participants were arranged in a supine position in a standard anatomical posture and instructed to remain calm for 10 minutes. Data were input into the VP-1000 arteriosclerosis detector as per protocol, with cuffs secured around the ankle arteries of both wrists adjusted to accommodate one finger’s width of tightness. Electrocardiogram electrodes and a heart sound amplification device were applied, followed by measurement of PWV for each subject.

### 2.6. Data analysis

The data were analyzed using SPSS 25.0 statistical software, and all measurement indicators were presented as mean ± standard deviation (*x* ± S). A paired sample *t* test was applied for normally distributed data, while a nonparametric test was used for data that did not follow a normal distribution. The analysis focused on evaluating changes in vascular elasticity, blood lipid levels, cardiac function, and other indicators before and after the intervention within the experimental group. Additionally, comparisons were made between the experimental and control groups. The significance level was set at α = 0.05, with a higher level of significance at α = 0.01.

## 3. Results

**Figure 3.**
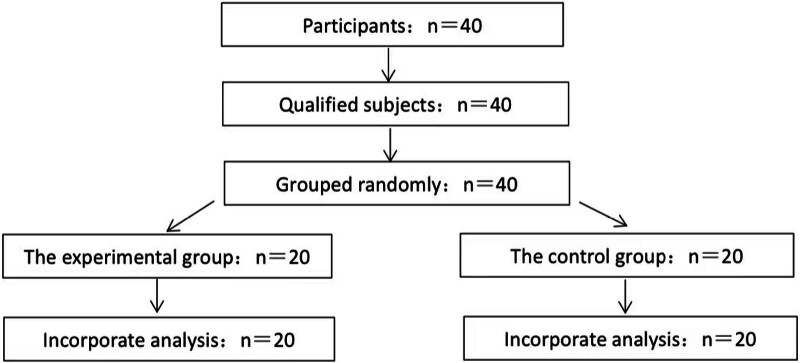
Grouping situation.

As shown in Figure 3, participants were recruited from May 2019 to July 2019 among volunteers from the Beijing Haidian District Health Qigong Association and surrounding communities of Beijing Sports University. A total of 40 volunteers who met the inclusion criteria were recruited, with 20 assigned to the experimental group and 20 to the control group. There were no dropouts among the participants during the course of the experiment. The average age of the participants was 61.78 ± 5.74 years. There were no difference in age, height, weight, or other general characteristics between the 2 groups (*P* > .05).

### 3.1. Vascular elasticity test results

Two parameters were evaluated: pulse wave velocity in the left ankle and right ankle. As shown in Table 2, the results demonstrated statistical differences in the left ankle (*P* = .02, *P* < .05) and right ankle (*P* = .00, *P* < .01) of the experimental group. This indicates that the vascular elasticity in the right ankle was significantly better than that in the left ankle, with a highly significant difference observed. Conversely, no differences were found in the control group (left ankle *P* = .084, *P* > .05; right ankle *P* = .08, *P* > .05).

**Table 2:** Change in the degree of vascular elasticity.

Parameters	Group	N	PWV (M ± SD)m/s		*P*
			Pre	Post	
Degree of vascular elasticity	Experience group (left ankle)	20	14.41 ± 3.10	13.33 ± 2.07	.02
	Control group (left ankle)	20	14.19 ± 1.83	14.87 ± 2.63	.08
	Experience group (right ankle)	20	14.22 ± 3.47	13.29 ± 2.84	.00
	Control group (right ankle)	20	14.57 ± 1.59	14.94 ± 1.59	.08

Following the exercise intervention, the experimental group showed significantly lower PWV compared to the control group (left ankle = 13.33 ± 2.07 m/s vs 14.87 ± 2.63 m/s, *P* = .045, *P* < .05; right ankle = 13.29 ± 2.84 m/s vs 14.94 ± 1.59 m/s, *P* = .007, *P* < .01).

### 3.2. Blood lipid test results

Table 3 indicates that following the intervention, significant differences were observed in triglyceride levels (TG) within the experimental group (*P* = .00, *P* < .01), whereas no changes were noted in the control group (*P* = .59, *P* > .05). Similarly, significant differences were found in total cholesterol (TC) within the experimental group (*P* = .00, *P* < .01), but not in the control group (*P* = .71, *P* > .05). There were also significant differences in high-density lipoprotein (HDL) levels between the 2 groups (*P* = .00, *P* = .01). Comparing mean values before and after the intervention, TG and TC decreased significantly in the experimental group, while HDL increased significantly, with a greater increase compared to the control group.

**Table 3:** Change in the blood fat.

Parameters	Group	N	Blood fat (M ± SD)mmol/L		*P*
			Pre	Post	
Blood fat	Experience group (TG)	20	1.93 ± 0.87	1.22 ± 0.40	.00
	Control group (TG)	20	1.95 ± 1.02	2.08 ± 0.89	.59
	Experience group (TC)	20	5.92 ± 0.79	4.56 ± 0.92	.00
	Control group (TC)	20	5.16 ± 0.92	5.26 ± 0.78	.71
	Experience group (HDL)	20	1.68 ± 0.20	2.54 ± 0.28	.00
	Control group (HDL)	20	1.65 ± 0.30	1.90 ± 0.32	.01

### 3.3. Cardiac function test results

Table 4 shows that following the intervention, there was a highly significant difference (*P* = .00, *P* < .01) in CO (Teich) within the experimental group, whereas no significant difference was observed in the control group (*P* = .71, *P* > .05). Additionally, there was a highly significant difference (*P* = .00, *P* < .01) in SV (Teich) in the experimental group, while no significant difference was found in the control group (*P* = .06, *P* > .05).

**Table 4:** Change in the cardiac function.

Parameters	Group	N	Cardiac function (M ± SD)CO(Teich)L/min;SV(Teich)mL		*P*
			Pre	Post	
Cardiac function	Experience group (CO(Teich))	20	3.97 ± 1.14	4.76 ± 0.88	.00
	Control group (CO(Teich))	20	4.69 ± 1.69	3.81 ± 1.01	.71
	Experience group (SV(Teich))	20	59.25 ± 11.62	67.05 ± 11.32	.00
	Control group (SV(Teich))	20	62.65 ± 23.49	56.05 ± 15.93	.06

## 4. Discussion

### 4.1. The effect of combining health Qigong with dance exercise on the vascular elasticity of middle-aged and elderly women

PWV indicates the stiffness of the central elastic artery and some surrounding muscular arteries, encompassing large blood vessels and their branches. By measuring PWV values, pathological changes in medium-sized arteries like coronary arteries can be promptly identified. Typically, higher PWV values indicate greater artery stiffness and reduced compliance. The progression of vascular diseases is gradual, and alterations in arterial wall elasticity underpin the onset and progression of various cardiovascular and cerebrovascular conditions. Therefore, diminished arterial elasticity has emerged as a critical indicator of cardiovascular disease risk factors.

Numerous studies have shown that prolonged engagement in traditional exercises such as Taijiquan and health Qigong can enhance vascular health and delay the aging process of blood vessels.^[17–19]^ Specifically, these exercises have been noted for their positive impact on the elasticity of blood vessels in middle-aged and elderly women, contributing to overall health improvement. In this study, an intervention program combining Health Qigong with Tibetan dance movements was implemented. Unlike repetitive movements, Tibetan dance involves diverse patterns including straight lines, arcs, and curves. Each movement incorporates dynamic transformations like stretching, opening and closing, and virtual and real movements, emphasizing rotational, and winding motions.

This exercise regimen mechanically massages blood vessels and lymphatic vessels, thereby preserving their elasticity. Our blood can be likened to water held within a towel, with blood vessels serving as transportation channels. Rotary movements exert greater force than simple downward pressure, aiding in alleviating circulation issues and spasms in small vessels. This action compels smaller vessel branches adjacent to blocked or narrow arteries to expand, facilitating venous blood return. Furthermore, whole-body muscle relaxation during exercise reflexively prompts vasodilation and reduces blood pressure, thereby easing the workload on the heart.

In conclusion, integrating Tibetan dance elements into Health Qigong enhances the elasticity of blood vessels in middle-aged and elderly women, aligning with the research hypothesis.

### 4.2. The effect of health Qigong combined with dance exercise on the blood fat of middle-aged and elderly women

As traditional sports become increasingly popular among the general population, their effectiveness in managing cardiovascular and cerebrovascular diseases has been increasingly validated. Regular practice of fitness Qigong aids in regulating blood lipid metabolism, lowering TC levels, and effectively preventing hyperlipidemia.^[20–22]^

The study’s findings revealed a significant decrease in TG and TC levels among participants in the experimental group after 12 weeks of health Qigong combined with dance exercise. Additionally, the level of HDL significantly increased, with a greater increase observed compared to participants who did not engage in the exercise program.

The significant improvement in blood lipid levels among our participants is believed to be primarily attributed to the inclusion of Tibetan dance movements in the health Qigong exercise program, which integrates a slightly simpler and slower pace, yet intensifies the overall routine compared to health Qigong alone. This adjustment maintains a manageable difficulty level, enhancing persistence and skill mastery among practitioners. The continuous coordination of hands and feet during exercise engages muscles, reduces body fat percentage, and enhances muscle strength. Regular adherence to this exercise regimen influences blood lipid composition and exerts an antioxidant effect. Increased blood lipids are absorbed, and HDL levels, known for their anti-atherosclerotic effects, are elevated, thereby effectively reducing blood lipid levels.

In summary, integrating Tibetan dance elements with Health Qigong effectively lowers TG and TC levels in the blood of middle-aged and elderly women. It also enhances HDL levels, reduces fat deposition in vascular walls, and helps prevent vascular atherosclerosis, as well as cardiovascular and cerebrovascular diseases. These outcomes align closely with the research hypothesis.

### 4.3. The effect of health Qigong combined with dance exercise on cardiac function of middle-aged and elderly women

The heart serves as the primary energy source for supplying the body’s metabolic needs. Insufficient pumping by the heart can lead to tissue and organ ischemia, potentially causing organ failure. Following 12 weeks of health Qigong combined with Tibetan dance exercise, significant improvements were observed in cardiac function indicators, specifically CO and SV, with highly significant differences noted (*P* = .00, *P* < .01). These parameters reflect myocardial contractility and overall heart function. CO relates to the volume of blood pumped by the ventricle per minute, while SV pertains to the volume of blood ejected from the ventricle in one heartbeat cycle. The study results indicated that regular health Qigong combined with dance exercise led to an increase in CO by 0.79 L/min and SV by 7.8 mL, contributing to an overall improvement in cardiac index. These findings demonstrate that health Qigong combined with dance exercise effectively enhances cardiac function, increases SV and cardiac blood volume, improves left ventricular pumping and ejection capabilities, and enhances overall blood circulation in the body. This ultimately enhances the efficiency of the cardiovascular system.

All types of physical exercise can positively impact the function of human internal organs, with traditional sports potentially enhancing the stroke output and stroke index of the heart.^[23,24]^ Increased CO can enhance aerobic metabolism in cardiac cells, bolster myocardial reserve capacity, mitigate cardiac dysfunction stemming from chronic diseases, and contribute to the stability of the cardiovascular system.^[25,26]^

The observed changes in the indicators are attributed to the gentle and regular exercise regimen practiced by participants, which emphasized remaining calm, relaxed, and focused. This exercise approach required gentle movements involving every joint and muscle. It effectively stimulated muscle contractions, enhancing overall blood circulation, coronary artery blood flow, and venous return flow. These improvements contributed to increased blood flow in cardio-cerebral vessels, thereby promoting metabolism. Simultaneously, the exercise regimen reduced sympathetic nerve excitability while enhancing vagus nerve excitability. This led to a decrease in heart rate and strengthened central functions related to vasodilation and blood pressure regulation. The regimen also promoted coronary collateral circulation, widened vascular lumens, enhanced wall elasticity, and improved myocardial blood flow. These benefits were achieved through practices like light, deep, and uniform abdominal breathing during the exercise.

This study has several limitations. Firstly, the PWV measurement relied on the VP-1000 arteriosclerosis detector, which may not be readily available in all centers. Secondly, the calculation of effective arterial elastance and left ventricular end-systolic elastance ratio, used to assess cardiovascular performance and cardiac energetics, was not included.^[27,28]^

In conclusion, integrating Tibetan dance elements into health Qigong improves heart function among middle-aged and elderly women, aligning with the research hypothesis.

## 5. Conclusion

This study integrated health Qigong with Tibetan dance to enhance arterial elasticity, blood lipid levels, and heart function among middle-aged and elderly women. After the exercise intervention, there were notable increases in cardiac pumping force, myocardial contraction strength, and SV, effectively enhancing vascular elasticity and blood circulation. Additionally, the intervention led to reductions in TG and TC levels, an increase in HDL, and a decrease in vascular wall fat deposition. These outcomes collectively contribute to the prevention of vascular atherosclerosis, cardiovascular diseases, and cerebrovascular diseases.

This study contributes to existing research by emphasizing the risks associated with declining cardiovascular function in middle-aged and elderly women. It also introduces new forms of exercise and proposes fresh research avenues. Based on its findings, combining health Qigong with Tibetan dance emerges as an effective method for preventing cardiovascular diseases.

## Acknowledgments

We would like to acknowledge the hard and dedicated work of all the staff that implemented the intervention and evaluation components of the study.

## Author contributions

**Conceptualization:** Qian-Yi Zhang, Hui Yang.

**Data curation:** Fan-Wen Zeng.

**Formal analysis:** Wei-Jie Zong, Xiang-Ji Peng.

**Funding acquisition:** Fan-Wen Zeng, Hui Yang.

**Methodology:** Wei-Jie Zong, Xiang-Ji Peng.

**Writing – original draft:** Qian-Yi Zhang, Hui Yang.

**Writing – review & editing:** Qian-Yi Zhang, Hui Yang.
